# Dosimetric comparison between jaw tracking and static jaw techniques in intensity-modulated radiotherapy

**DOI:** 10.1186/s13014-015-0329-4

**Published:** 2015-01-27

**Authors:** Zhongsu Feng, Hao Wu, Yibao Zhang, Yunjun Zhang, Jinsheng Cheng, Xu Su

**Affiliations:** Chinese Center for Medical Response to Radiation Emergency, National Institute for Radiological Protection, Chinese Center for Disease Control and Prevention, 2 Xinkang Street, Deshengmenwai, Beijing, 100088 China; Key laboratory of Carcinogenesis and Translational Research (Ministry of Education), Department of Radiotherapy, Peking University Cancer Hospital & Institute, 52 Fuchen Road, Haidian, Beijing, 100142 China; School of Foundational Education, Peking University Health Science Center, 38 Xueyuan Road, Haidian, Beijing, 100191 China

**Keywords:** Jaw tracking, IMRT, Dosimetry, OARs sparing

## Abstract

**Purpose:**

To compare the dosimetric differences between jaw tracking technique (JTT) and static jaw technique (SJT) in dynamic intensity-modulated radiotherapy (d-IMRT) and assess the potential advantages of jaw tracking technique.

**Methods:**

Two techniques, jaw tracking and static jaw, were used respectively to develop the d-IMRT plans for 28 cancer patients with various lesion sites: head and neck, lungs, esophageal, abdominal, prostate, rectal and cervical. The dose volume histograms (DVH) and selected dosimetric indexes for the whole body and for organs at risk (OARs) were compared. A two dimensional ionization chamber Array Seven29 (PTW, Freiburg, Germany) and OCTAVIUS Octagonal phantom (PTW, Freiburg, Germany) were used to verify all the plans.

**Results:**

For all patients, the treatment plans using both techniques met the clinical requirements. The V_5_, V_10_, V_20_, V_30_, V_40_ (volumes receiving 5, 10, 20, 30 and 40 Gy at least, respectively), mean dose (D_mean_) for the whole body and V_5_, V_10_, V_20_, D_mean_ for lungs in the JTT d-IMRT plans were significantly less than the corresponding values of the SJT d-IMRT plans (p < 0.001). The JTT d-IMRT plans deposited lower maximum dose (D_max_) to the lens, eyes, brainstem, spinal cord, and right optic nerve, the doses reductions for these OARs ranged from 2.2% to 28.6%. The JTT d-IMRT plans deposited significantly lower D_mean_ to various OARs (all p values < 0.05), the mean doses reductions for these OARs ranged from 1.1% to 31.0%, and the value reductions depend on the volume and the location of the OARs. The γ evaluation method showed an excellent agreement between calculation and measurement for all techniques with criteria of 3%/3 mm.

**Conclusions:**

Both jaw tracking and static jaw d-IMRT plans can achieve comparable target dose coverage. JTT displays superior OARs sparing than SJT plans. These results are of clinical importance, especially for the patients with large and complex targets but close to some highly radio-sensitive organs to spare, and for patients with local recurrent or secondary primary malignant lesion within a previously irradiated area.

## Introduction

Nowadays, dynamic intensity-modulated radiotherapy (d-IMRT) is widely used in the treatment of cancer patients. Compared with three dimensional conformal radiotherapy, d-IMRT allows to increase the dose conformity of the target while decreasing normal tissue doses [1].

In dynamic IMRT plans, the multi-leaf collimators (MLCs) translate continuously at variable speeds during the irradiation while the upper and lower jaws stay static. According to the study by LoSasso et al. [2], the MLC transmission increases with increasing jaw field size and beam energy. Cadman et al. [3] found that the transmission through the jaw and the MLC together is smaller than 0.1%. Jaw tracking technique provided by linear accelerators keeps jaws during dose delivery as close as possible to the MLC aperture, and further minimizes leakage and transmission through the MLC leaves. Joy et al. [4] evaluated the dosimetric effects of jaw tracking in step-and-shoot IMRT, but failed to indicate which patients would benefit most from jaw tracking. The dosimetric benefits of jaw tracking for prostate and head and neck (H&N) patients using d-IMRT and volumetric-modulated arc therapy (VMAT) were also evaluated by others [5,6]. It showed that, the organs far from the target showed larger sparing in jaw-tracking static arc than the organs adjacent to the target. TrueBeam (Varian Medical Systems, Palo Alto, CA) is a new generation of linear accelerator providing jaw tracking technique (JTT), which traces the MLC aperture with jaws to minimize the leakage and transmission of the MLC leaves, hence further reduces the OAR doses adjacent to the target, and potentially improves the dose fall-off towards the surrounding critical structures.

The main purpose of this study is to assess the potential advantages of the JTT provided by TrueBeam accelerators in reducing the doses to the organs at risk (OARs) while preserving adequate target dose coverage, and to evaluate which patients and which OARs would benefit most from JTT plans.

## Materials and methods

### Ethical consideration and consent

The use of the radiotherapy database for retrospective research has been approved by the committee of the Ethical Review Board of the Chinese Center for Disease Control and Prevention (No. LLSC2014-005). This research was waivered informed consent.

### Patient characteristics

Twenty eight cancer patients (median age 58 years, range 31–77 years) treated with radiotherapy in our department were retrospectively included in this study. The lesion distributions of the selected cases included head and neck, lungs, esophageal, abdominal, prostate, rectal and cervical, as shown in Table 1. One local recurrent nasopharyngeal carcinoma (NPC) patient was included in the H&N group.

**Table 1:** **Lesion distributions and treatment characteristics of the selected cases**

**Lesion sites**	**n**	**Total dose (Gy)**		**PTV (cm** ^**3**^ **)**	
		**GTV **	**CTV **		
		**Mean dose (range)**	**Mean dose (range)**	**Mean**	**SD**
Head and neck	6	66.4(60–70)	55.8(45–60)	693.38	168.8
Lungs	5	60.7(56–66)	54.9(50.6-60)	270	50.07
Esophageal	4	65(64,66)	50	536.28	39.4
Abdominal	3	53.5(50–60)	46(45–48)	454.1	181.24
Prostate	2	72	65(60,70)	174.85	35.75
Rectal	6	50.6	41.8	1163.57	55.14
Cervical	2	-	47.5(45,50)	1204.05	139.45

Abbreviations: *SD* standard deviation.

### Contouring of targets and OARs

All patients were immobilized with custom-made thermoplastic mask and underwent computed tomography (CT) scan with slice thickness of 3 mm (for head and neck patients) or 5 mm (for thoracic, abdominal and pelvic patients ) using the Siemens Somatom Sensation Open 40 slice CT scanner (Siemens, Forchheim, Germany). The targets and OARs were contoured slice by slice on the treatment planning CT images. Relevant organs were delineated for different anatomic regions. The body was segmented automatically by the Eclipse (Versions 11.0) Treatment Planning System (TPS) (Varian Medical Systems, Palo Alto, CA). Isotropic margins of 3 mm (for head and neck patients) or 5 mm (for thoracic, abdomen and pelvic patients) were created around the clinical target volume (CTV) and gross tumor volume (GTV) to generate the planning target volume (PTV) and planning gross tumor volume (PGTV), respectively.

### Treatment planning

The static jaw technique (SJT) d-IMRT plans were created using the Eclipse TPS using sliding window dynamic delivery and fixed beam angles. The selected energies were 6 MV (for head and neck patients) and 10 MV (for thoracic, abdominal and pelvic patients) respectively from a TrueBeam linear accelerator. The collimator angles were optimized according to the target shape. After IMRT optimization using the dose volume optimizer algorithm (version 11.0.31), the volumetric doses were calculated using the anisotropic analytical algorithm (version 11.0.31) with a dose calculation grid of 2 mm. The reference volume for the treatment planning was PTV. The normal tissue dose volume constraints used for the treatment planning are listed in Table 2. For all patients, the dose distributions met the planning requirements, i.e. at least 95% of the PTV received the prescribed dose, while the doses to the surrounding tissues were minimized. To create JTT d-IMRT plans, the SJT d-IMRT plans were duplicated and jaw tracking function was selected when calculating the leaf motions and volumetric doses. All the machine parameters and optimization parameters were identical to those of the SJT d-IMRT plans. The JTT plans reduce the dose leakage and transmission through the MLCs, which required renormalization to achieve the same PTV coverage as in the SJT d-IMRT plans.

**Table 2:** **Dose constraints used in treatment planning**

**Organ**	**Dose volume parameters**	**Organ**	**Dose volume parameters**
Lens	D_max_ < 7Gy	Heart	D_mean_ < 26Gy
Eye	D_max_ < 50Gy		V_30_ < 50%
	D_mean_ < 35Gy	Liver	D_mean_ < 30Gy
Optic nerve	D_max_ < 54Gy		1/3 volume of liver avoid irradiation
Brain stem	D_max_ < 54Gy	Kidney	D_mean_ < 18Gy
Parotid	V_30_ < 50%		V_12_ < 55%
	D_mean_ < 26Gy		V_20_ < 32%
Spinal cord	D_max_ < 40Gy	Intestine	V_40_ < 30%
Esophagus	V_35_ < 50%	Bladder	V_30_ < 50%
	D_mean_ < 34Gy	Femoral Head	V_20_ < 50%
Lung	V_5_ < 70%		V_30_ < 15%
	V_10_ < 50%	Rectum	V_30_ < 50%
	V_20_ < 30%		V_60_ < 35%
	D_mean_ < 15Gy		V_70_ < 20%

### Plan evaluation

The DVHs and the isodose curves of the JTT plans were compared with that of the SJT plans. The dose changes to the whole body were evaluated using V_5_, V_10_, V_20_, V_30_, V_40_, D_max_ and D_mean_ as listed in Table 3. The parameters of V_5_, V_10_, V_20_, D_max_, and D_mean_ were used to evaluate the dose changes to the lungs, as shown in Table 4. The dosimetric disparities of OARs were evaluated using the maximum dose (D_max_) and mean dose (D_mean_) as listed in Table 5 and Table 6.

**Table 3:** **Comparison of the whole body doses between the two techniques (** $\overline{\mathrm{X}}\pm \mathrm{s}$ **)**

**Body**	**SJT**	**JTT**	**p**
V_5_ (%)	37.2 ± 19.1	36.1 ± 18.6	<0.001
V_10_ (%)	29.0 ± 16.5	28.2 ± 16.0	<0.001
V_20_ (%)	19.9 ± 12.9	19.3 ± 12.4	<0.001
V_30_ (%)	12.0 ± 9.6	11.6 ± 9.2	<0.001
V_40_ (%)	7.6 ± 7.0	7.5 ± 6.9	<0.001
D_max_(Gy)	60.3 ± 8.5	60.5 ± 8.8	0.071
D_mean_(Gy)	9.8 ± 6.0	9.6 ± 5.8	<0.001

**Table 4:** **Comparison of lung doses between the two techniques (** $\overline{\mathrm{X}}\pm \mathrm{s}$ **)**

**Lung**	**SJT**	**JTT**	**p**
V_5_ (%)	48.3 ± 6.7	45.7 ± 5.9	<0.001
V_10_ (%)	34.9 ± 6.2	33.6 ± 5.8	<0.001
V_20_ (%)	20.5 ± 5.5	19.9 ± 5.1	<0.001
D_max_(Gy)	59.9 ± 6.4	60.00 ± 6.5	0.155
D_mean_(Gy)	11.4 ± 2.6	11.0 ± 2.5	<0.001

**Table 5:** **Comparison of OARs D**_**max**_ **between the two techniques (** $\overline{\mathrm{X}}\pm \mathrm{s}$ **)**

**OARs D**_**max**_ **(Gy)**	**SJT**	**JTT**	**p**
Right lens	5.6 ± 2.2	4.00 ± 1.7	0.031
Left lens	5.7 ± 1.6	4.2 ± 1.3	0.031
Right eye	26.3 ± 17.3	24.5 ± 17.8	0.031
Left eye	26.6 ± 13.9	24.9 ± 14.9	0.031
Right optic nerve	44.3 ± 8.9	43.4 ± 8.7	0.031
Left optic nerve	41.8 ± 10.8	40.8 ± 12.0	0.156
Brain stem	39.1 ± 12.0	37.2 ± 12.9	0.016
Right parotid	62.8 ± 10.9	63.2 ± 11.4	0.063
Left parotid	63.2 ± 7.3	63.6 ± 7.4	0.156
Spinal cord	31.0 ± 8.0	30.3 ± 7.9	<0.001
Esophagus	62.9 ± 5.5	62.8 ± 5.5	0.500
Heart	57.9 ± 5.9	57.9 ± 6.0	0.527
Liver	49.2 ± 6.1	49.2 ± 6.1	0.094
Right kidney	36.3 ± 15.9	36.3 ± 16.0	0.406
Left kidney	37.2 ± 9.3	37.0 ± 9.1	0.406
Intestine	50.6 ± 3.7	50.7 ± 3.9	0.313
Bladder	56.1 ± 9.0	56.2 ± 8.9	0.156
Femoral head	39.7 ± 5.2	39.8 ± 5.3	0.156
Rectum	60.6 ± 8.9	60.2 ± 9.4	0.156

**Table 6:** **Comparison of OARs D**_**mean**_ **between the two techniques (** $\overline{\mathrm{X}}\pm \mathrm{s}$ **)**

**OARs D**_**mean**_ **(Gy)**	**SJT**	**JTT**	**p**
Right lens	4.9 ± 2.0	3.38 ± 1.44	0.031
Left lens	5.0 ± 1.4	3.59 ± 1.07	0.031
Right eye	7.1 ± 3.8	5.56 ± 3.55	0.031
Left Eye	7.7 ± 2.6	5.99 ± 2.83	0.031
Right optic nerve	25.5 ± 12.4	23.87 ± 13.33	0.031
Left optic nerve	26.0 ± 11.1	24.37 ± 12.26	0.031
Brain stem	23.7 ± 13. 5	22.03 ± 14.08	0.016
Right parotid	29.3 ± 4.9	27.41 ± 4.02	0.031
Left parotid	28.7 ± 4.1	27.23 ± 3.46	0.031
Spinal cord	19.0 ± 8.0	18.1 ± 7.6	<0.001
Esophagus	29.4 ± 8.4	28.9 ± 8.1	0.016
Heart	17.9 ± 11.9	17.5 ± 11.8	0.004
Liver	12.2 ± 9.9	11.9 ± 9.8	0.031
Right kidney	8.5 ± 4.1	8.2 ± 4.0	0.031
Left kidney	6.6 ± 4.8	6.3 ± 4.5	0.031
Intestine	27.4 ± 3.4	27.1 ± 3.4	0.016
Bladder	33.5 ± 8.7	33.1 ± 8.5	0.004
Femoral head	14.1 ± 1.6	13.7 ± 1.5	0.004
Rectum	35.8 ± 4.5	35.2 ± 4.7	0.031

### Plan verification

The Eclipse TPS was used to create plans for verification by converting the SJT and JTT plans to OCTAVIUS Octagonal phantoms (PTW, Freiburg, Germany). Then the volumetric doses were re-calculated, and the dose distributions in effective measurement points of the two dimensional ionization chamber Array Seven29 (PTW, Freiburg, Germany) were exported for all plans. By using the γ evaluation method of VeriSoft software (version 5.1) (PTW, Freiburg, Germany), the measured dose planes were compared with the computed dose distribution using criteria of 3% dose difference and 3 mm distance to agreement.

### Statistical analysis

The data were presented as the averages of all patients followed by the standard deviation ($\overline{\mathrm{X}}\pm \mathrm{s}$). The PASW Statistics (version 18.0) software (SPSS; Chicago, IL, USA) was applied for statistical analysis. To compare the results of the two techniques, Wilcoxon Signed-Ranks test was performed with p values <0.05 considered as significant.

## Results

**Figure 1:**
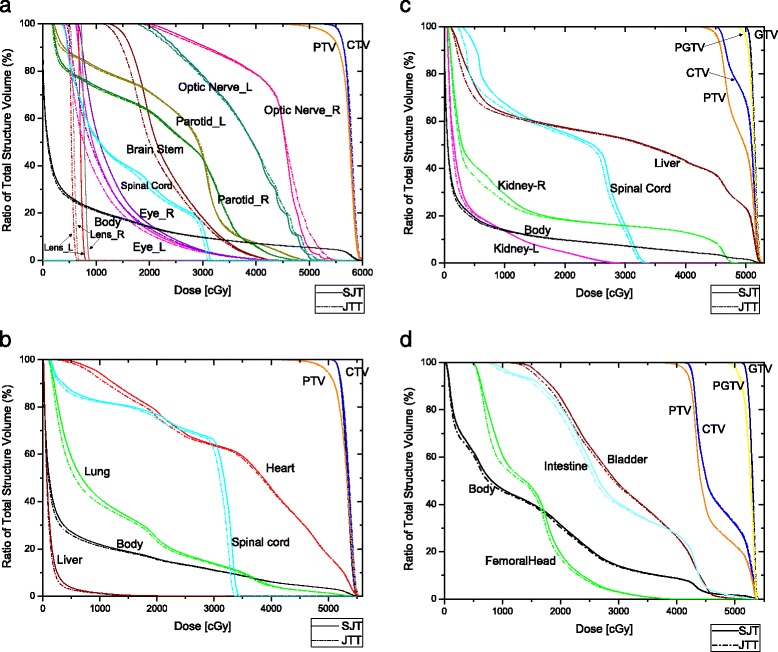
**DVH comparison between SJT and JTT plans for different body sites: (a) H&N, (b) Thoracic, (c) Abdominal, (d) Pelvic case.** CTV: Clinical target volume; PTV: Planning target volume; GTV: Gross tumor volume; PGTV: Planning gross tumor volume.

As shown in the DVH comparison (Figure 1), JTT d-IMRT plans reduce doses to the normal tissues, especially in the low dose regions.

Table 3 lists the comparison of the whole body doses between the two techniques. The JTT d-IMRT plans displayed significantly lower percentages of V_5_, V_10_, V_20_, V_30_, V_40_ and significantly lower mean doses of the whole body (p < 0.001). There was no significant difference of maximum doses delivered to the whole body (p = 0.071).

Table 4 lists the statistical data of the lungs for all thoracic cases. The V_5_, V_10_, V_20_, D_mean_ of lungs in JTT d-IMRT plans are significantly lower than the corresponding values of the SJT d-IMRT plans (p < 0.001), the mean reduction was 2.6%, 1.3%, 0.6% and 0.4%, respectively. There was no significant difference of maximum doses delivered to the lungs (p = 0.155).

This study demonstrates that the mean values of the maximum doses to the lens, eyes, brainstem, spinal cord, and right optic nerve are significantly reduced in the JTT plans (all p values <0.05), as shown in Table 5. The doses reduction for these OARs ranged from 2.2% to 28.6%, and the maximum dose to lens is reduced most obviously.

Table 6 lists the mean doses to various OARs for all patients. The JTT d-IMRT plans give significantly lower mean doses to the studied OARs (all p values <0.05). The mean doses reduction for these OARs ranged from 1.1% to 31.0%, and the reduction values are depend on the volume and the location of the OARs.

Treatment plan verification of the two techniques is generally performed with the γ evaluation method. The γ was compared locally, and the γ pass rate for SJT and JTT is 93.9 ± 1.98 and 94.1 ± 1.95, respectively. The comparison of γ between the two techniques showed no statistical difference (p = 0.132). Dose calculation and delivery are both accurate for SJT and JTT plans at criteria of 3%/3 mm using γ analysis.

## Discussion

Jaw tracking methods have also been used in other treatment modalities aiming to achieve better target coverage and critical structure sparing. Joy et al. [4] evaluated the dosimetric effects of jaw tracking in step-and-shoot IMRT, showing an overall reduction of normal tissue doses. Most patients had reductions of V_5_, V_10_, and V_20_ by less than 2% in the normal tissues. Schmidhalter et al. [5] evaluated the leaf transmission reduction using moving jaws in dynamic MLC IMRT, and demonstrated that the undesired doses to the body volume minus the planning target volume decreased by up to 1.8% and 1.5% for prostate and H&N patients. Simultaneously, the MU increased by up to 3.1% and 2.8%, respectively. Kim et al. [6] assessed the potential advantages of jaw tracking technique by using control point sequences of VMAT planning, showing that, for H&N cases, the mean dose reductions for all the OARs ranged from 4.3% to 11.9%, and for prostate patients, the organs distant from the target were spared better in jaw-tracking static arc (JTSA) plans. The dose reductions were more significant in the dose regions of D_80_, D_90_ and D_95_ for all the patients in JTSA plans.

However, previous studies [4-6] included no abdominal cases or other pelvic lesion except prostate: the number of OARs was less than that of this study. Moreover, they didn’t indicate which cases and which organs would benefit most from JTT.

This study selected cases of various body sites. For large and complex targets, such as NPC, the large target volumes are surrounded by many critical neural tissues and sensitive structures such as lens, optic nerves, brain stem, parotid glands and spinal cord. This study demonstrates that the mean values of the maximum doses to the lens, eyes, brainstem, spinal cord, and right optic nerve are significantly reduced in the JTT plans (all p values <0.05) as shown in Table 5. Perhaps because these OARs do not overlap with the target in the selected cases, yet the left optic nerve and other OARs are partially or wholly covered by PTVs. The reduction of the maximum dose to OARs may reduce the risk of radiation injury [7,8], such as radiation induced myelitis and cataract. It is also more meaningful for the radiotherapy patients with local recurrent or second primary malignant lesion within or adjacent to a previously irradiated area.

The JTT technique also significantly reduces the low dose regions. In the thoracic cases, the JTT technique significantly reduces the V_5_, V_10_, V_20_ to the lungs, as shown in Table 4, compared to the SJT plan (p < 0.001). The results may mostly benefit Hodgkin’s lymphoma patients whose lungs are largely within the treatment fields during radiotherapy. The JTT technique may reduce the risk of pulmonary toxicity since radiation pneumonitis rate is correlated with low dose volumes [9-12]. The risk of radiation induced secondary malignancies is another concern, which is strongly associated with low dose exposure of normal tissues during IMRT [13].

In addition, this study suggests that the JTT plans give significantly lower mean doses to various OARs (all p values <0.05, refer to Table 6 for details). The JTT associated OAR dose reductions are more obvious for the mean doses to the structures of small volume (such as lens) or structures far from the targets (such as left optic nerve, esophagus, and kidneys in this study). The mean doses of OARs have been widely used to predict the probability of radiation toxicity [9-12], such as pulmonary toxicity. Therefore, JTT plans may decrease the probability of developing late side effects or secondary neoplasm.

Some problems exist in the current jaw tracking technique though. Firstly, jaw tracking is not considered in the optimization of JTT d-IMRT or in the step-and-shoot jaw tracking IMRT plans [4], as limited by the optimization algorithm of TPS. However, the Eclipse TPS (version 11.0) provides jaw tracking option in the VAMT plan optimization, which is applicable on Varian Truebeam accelerators. Secondly, the output factor of the accelerator varies dramatically in jaw tracking technique. Usually, the output factors are measured on the central axis of the beam. However, there are many off-axis fields in jaw tracking plans, which would affect the output factor and should be considered in treatment planning. Joy et al. [4] calculated the off-axis output factors and demonstrated decreases of 1% to 3% when shifting away from central axis. Although all the SJT and JTT plans in this study can pass the dosimetric verification, this issue was not solved so far and further research is needed. Thirdly, the performance of flattening filter-free (FFF) module combined with the jaw tracking technique of TrueBeam accelerator is still uncertain. There were several reports [14-19] about the clinical usage of FFF beams, indicating more potential of reducing the doses to OARs with comparable target dose coverage. The potential benefit of applying jaw tracking technique in FFF beams will be further studied in the future work.

## Conclusions

This study demonstrates that all the SJT and JTT plans could meet the clinical objectives and are clinically applicable. The dose measurements agreed well with TPS calculated doses. JTT displays superior OARs sparing than SJT plans. These results are of clinical importance, especially for the patients with large and complex targets but close to some highly radio-sensitive organs to spare (such as lens or gonad), and for patients with local recurrent or secondary primary malignant lesion within a previously irradiated area. More studies on off-axis output factors and combined application of FFF module should be conducted in the future.

## Abbreviations

JTT: Jaw tracking technique
SJT: Static jaw technique
d-IMRT: dynamic Intensity-modulated radiotherapy
DVH: Dose-volume histogram
OARs: Organs at risk
V_x_: Percentage volume of organs receiving at least the x Gy
MLC: Multi-leaf collimator
VMAT: Volumetric-modulated arc therapy
H&N: Head and neck
NPC: Nasopharyngeal carcinoma
CT: Computed tomography
TPS: Treatment planning system
CTV: Clinical target volume
PTV: Planning target volume
GTV: Gross tumor volume
PGTV: Planning gross tumor volume
SD: standard deviation
JTSA: Jaw-tracking static arc
LMC: Leaf motion calculator
FFF: Flattening filter-free
